# Astrocytic Ca^2+^ Waves Guide CNS Growth Cones to Remote Regions of Neuronal Activity

**DOI:** 10.1371/journal.pone.0003692

**Published:** 2008-11-12

**Authors:** Johanna Hung, Michael A. Colicos

**Affiliations:** Department of Physiology and Biophysics and the Hotchkiss Brain Institute, University of Calgary, Calgary, Alberta, Canada; The Rockefeller University, United States of America

## Abstract

Activity plays a critical role in network formation during developmental, experience-dependent, and injury related remodeling. Here we report a mechanism by which axon trajectory can be altered in response to remote neuronal activity. Using photoconductive stimulation to trigger high frequency action potentials in rat hippocampal neurons *in vitro*, we find that activity functions as an attractive cue for growth cones in the local environment. The underlying guidance mechanism involves astrocyte Ca^2+^ waves, as the connexin-43 antagonist carbenoxolone abolishes the attraction when activity is initiated at a distance greater than 120 µm. The asymmetric growth cone filopodia extension that precedes turning can be blocked with CNQX (10 µM), but not with the ATP and adenosine receptor antagonists suramin (100 µM) and alloxazine (4 µM), suggesting non-NMDA glutamate receptors on the growth cone mediate the interaction with astrocytes. These results define a potential long-range signalling pathway for activity-dependent axon guidance in which growth cones turn towards directional, temporally coordinated astrocyte Ca^2+^ waves that are triggered by neuronal activity. To assess the viability of the guidance effect in an injury paradigm, we performed the assay in the presence of conditioned media from lipopolysaccharide (LPS) activated purified microglial cultures, as well as directly activating the glia present in our co-cultures. Growth cone attraction was not inhibited under these conditions, suggesting this mechanism could be used to guide regeneration following axonal injury.

## Introduction

The path that axons follow is fundamental in defining neuronal connectivity in the brain. Hippocampal pyramidal neurons have long axons that branch and form thousands of *en passant* and *bouton* synapses [1]. Since axons span longer distances than dendrites and can therefore target diverse areas of the brain, axon guidance is a form of structural change that is potentially more powerful than mechanisms involving remodelling dendritic spines or subtle shifting of synaptic strengths [2], [3]. Convincing evidence that axons in some regions of the adult brain exhibit considerable organizational plasticity comes from animal and human studies involving injury to the peripheral nervous system, stroke, or amputation of digits and limbs. After such injury there is massive reorganization in sensory-motor areas that involve axonal rewiring in the cortex, thalamus and spinal cord [4]. Axon remodeling also occurs following traumatic and ischemic injuries in the central nervous system [5]. To achieve functional recovery after injury, axons must navigate their way back to their proper targets or find new ones. However, a caveat of axon regeneration is that glial cells secrete proinflammatory cytokines and other factors in response to injury, and these factors can impair axon growth in the injured region [6]–[8]. Some types of central axons maintain the capacity for growth in the adult brain even in the absence of injury. Recent implementations of *in vivo* multi photon based time-lapse imaging technologies have observed cell-type specific changes in axon morphology in the neocortex of normal adult mice [2].

Changes in connectivity that go beyond simple refinement of existing connections require axon branching and outgrowth, processes that can be modulated by activity [4]. Evidence for several types of activity related axon growth mechanisms have been found. For example, previous studies have found that action potential-induced depolarization of the growth cone membrane [9]–[11], and transient physiological electric fields in the vicinity of growth cones [12] can affect directional guidance. Growth cones also respond to focal gradients of neurotransmitters like adenosine, glutamate, acetylcholine and serotonin [13]–[16].

Bidirectional communication systems between neurons and astrocytes have recently been established as an important point of regulation of neuronal function on many levels, including neuronal growth [17]. Although astrocytes are not excitable, recent findings indicate they may play a role in rapid signal transmission in the brain. Astrocytes are equipped with glutamate-sensitive ion channels that are activated by excitatory neuronal activity, causing immediate and transient cytoplasmic Ca^2+^ elevations [18]. These Ca^2+^ elevations propagate into adjacent astrocytes as intercellular Ca^2+^ waves by intercellular passage of second messengers through gap junctions [18]–[20]. In response to elevated cytoplasmic Ca^2+^, astrocytes release glutamate, ATP, and D-serine into the extracellular space [20]–[22]. It is currently unknown whether temporal and spatial chemical gradients created by astrocytic Ca^2+^ waves could be detected by growth cones and used as a directional cue.

Here we went beyond focal neurotransmitter gradient experiments to see how growth cones would respond to actual physiological stimulation of spatially displaced neurons. We used photoconductive stimulation, a non-invasive method of triggering high frequency action potentials in individual cultured hippocampal neurons [23]–[25]. The strength of this stimulation technology is that it is non-invasive, requiring no physical perturbation to the cell with electrodes. An added advantage is that it has greater spatial specificity than extracellular field stimulation and greater temporal resolution than direct neurotransmitter application. This technique has been previously used to demonstrate activity-driven new synapse formation [24], and allows the dynamic observation of the unperturbed cellular elements within a neuron. We found that growth cones were attracted towards regions of activity and astrocytic Ca^2+^ wave propagation was necessary for growth cone turning when neuronal activity was initiated from more than one cell length away. Asymmetric growth cone filopodia extension preceded turning, a process dependent mechanistically on extracellular Ca^2+^ levels and correlated with redistribution of the SNARE protein VAMP-2. VAMP-2 is a key molecule for membrane expansion at the tips of growing neurites [26], [27] so its involvement further implicates the observed filopodia asymmetry as a mechanism for directional growth. The effect was blocked by the AMPA/KA receptor antagonist 6-cyano-7-nitroquinoxaline (CNQX), implicating non-NMDA glutamate receptors in the mechanism.

These results demonstrate a novel CNS axon guidance system for directing axon growth to regions of neuronal activity. To investigate the possibility that this mechanism could be used to control axon growth after injury, we performed our experiments in conditioned media from lipopolysaccharide (LPS)-activated human adult microglia cultures and rat hippocampal cultures. We observed that growth cones could still be guided by activity in these conditions, suggesting glial wave induction may be a viable mechanism for controlling axon growth after injury.

## Results

### Photoconductive stimulation of neurons triggers astrocyte Ca^2+^ waves

Photoconductive stimulation is a technique that can initiate action potentials in neurons with high spatial and temporal resolution [23]–[25], resulting in neuronal activity that is indistinguishable from spontaneously occurring action potentials (Figure S1). Utilizing a capacitive charge build-up on the surface of silicon, the region of stimulation is spatially defined by the diameter of the photocurrent induced from a beam of light.

**Figure 1:**
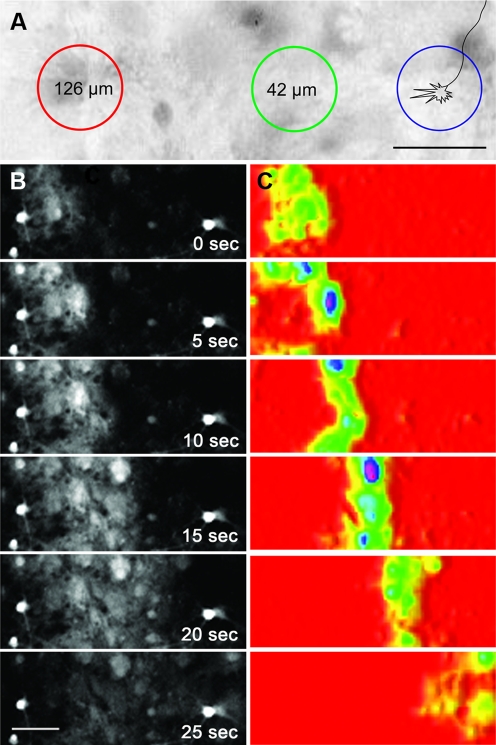
Local calcium elevation in astrocytes leads to the propagation of an intercellular Ca^2+^ wave. Calcium levels were monitored using fluo-4 AM. (a) Illustration of the experimental protocol, which entailed the depolarization of a 42 µm diameter area either 42 µm or 126 µm to the right of an axonal growth cone. (b,c) Representative images of a glial wave. Panel c shows the progression of the wave front after image subtraction in ImageJ (W. Rasband, NIH). Scale bars, 50 µm.

The spatial dynamics of intracellular Ca^2+^ in neurons and astrocytes were monitored using Fluo-4 AM, a fluorescent calcium indicator. Fig. 1b,c and Video S1 show an example of an astrocyte Ca^2+^ wave after stimulation. The image sequences were captured at low magnification (10×) to demonstrate the nature of the wave. Subsequent experiments required the use of a higher magnification (100×) objective to monitor growth cone behaviour. Glial waves fronts moved at an average speed of 7 µm/sec, ranging from 2 to 17 µm/sec (calculated from 10 waves from 5 experiments).

### Growing pyramidal cell axons are attracted to regions of neuronal activity

Our experimental protocol involved the stimulation of a ∼42 µm diameter area whose proximal edge was either ∼42 µm or ∼126 µm from the right side of a specific axonal growth cone, approximately 90° from the initial direction of axon extension (Fig. 1a). The ‘right side’ was defined from the perspective of the trailing axon. Stimulation induced neuronal activity and glial waves were triggered from 42 µm away to examine guidance effects in the absence of direct growth cone depolarization. Cultures were transfected with YFP-actin for visualization of a defined population of growth cones during live cell imaging. Axonal growth cones were identified based on morphology [1, See Methods]. The accuracy of this method was confirmed by post-experiment immunostaining for the axonal marker tau-1. Out of the 17 growth cones on which experiments were performed, 12 could be found post fixation. Of these 12 morphologically identified axons, 11 were positive for tau-1.

**Figure 2:**
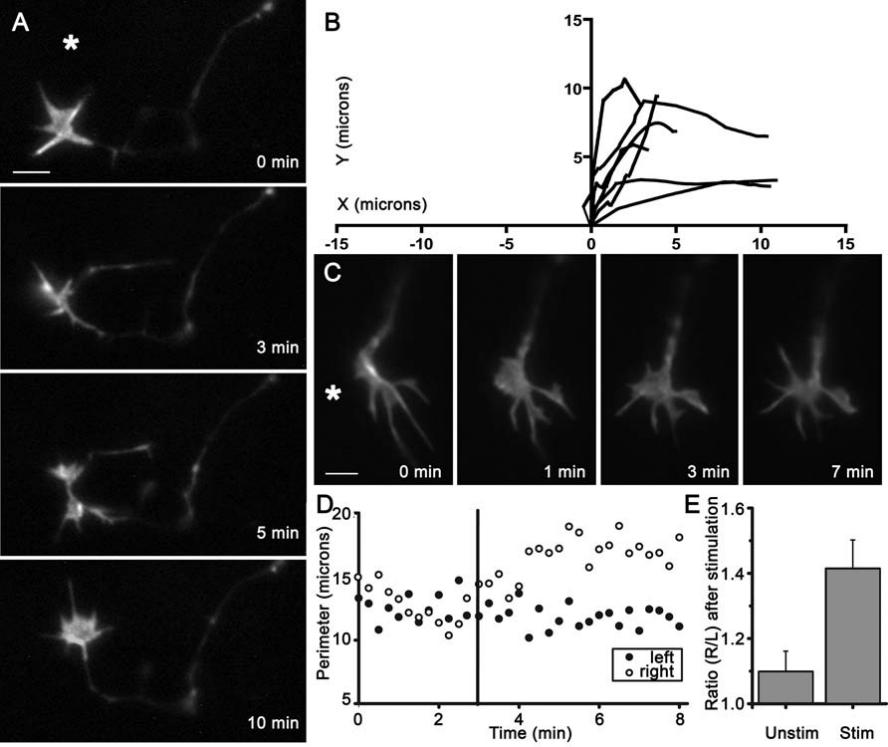
Activity induces attractive growth cone turning and filopodia sprouting. (a) An example of a growth cone that turned when the stimulation was repeated once every 5 minutes, as visualized by YFP-actin. (b) Superimposed traces of the trajectories of growth cones (n = 5) that extended more than 5 µm during long term experiments wherein neurons to the right side of the growth cone were stimulated every 5 minutes for 0.5 to 1 hour. The origin shows the center of the growth cone at t = 0, when its direction of growth was vertical. The axes show the distance in microns. (c) More filopodia protrude on the side of the growth cone that faces the area of stimulation, as visualized by YFP-actin. (d) A representative time series of the total perimeter of the left and right side of a growth cone during an experiment, measured from images taken 20 seconds apart. The vertical bar marks the first set of images taken after the stimulation. (e) The ratio of the total perimeter of the right side to that of the left (R/L) after a real stimulation is greater compared with that of a mock stimulation (unstimulated, n = 25; stimulated, n = 20). Bars show the mean, error bars are the SEM. Scale bars, 2 µm.

**Figure 3:**
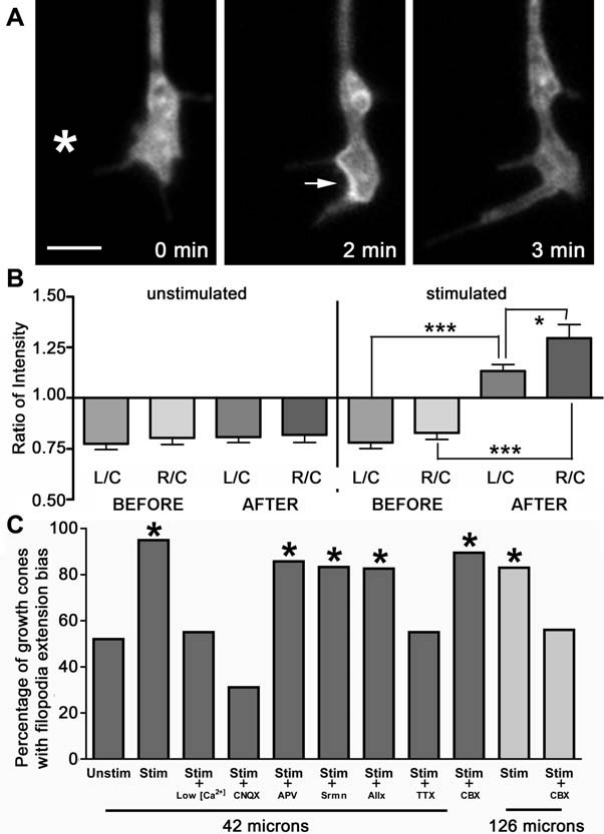
A role for VAMP-2, calcium, non-NMDA receptors and gap junctions in activity-induced attractive filopodia extension. (a) 2 minutes after stimulation GFP-VAMP-2 clusters near the membrane, mostly on the right side. (b) The mean grey value (intensity) of a region of interest was measured from the left side (L), right side (R) and cytoplasm (C) of growth cones from GFP-VAMP-2 neurons before and after stimulation. The intensity ratios L/C and R/C increase after a stimulation (n = 20; p<0.0001, two-sided unpaired student's t-test). R/C is also significantly higher then L/C after stimulation (p = 0.0344). The intensity ratios (L/C and R/C) do not change when cells are monitored for the same amount of time without a voltage pulse (n = 21, p>0.05). Error bars represent standard deviations. (c) The attractive filopodia extension is calcium dependent and is blocked by CNQX. The effect is blocked by CBX when depolarization is performed at 126 µm but not 42 µm. Control, n = 25; stimulated (42 µm), n = 20; low Ca^2+^ (42 µm), n = 20; 10 µM CNQX(42 µm), n = 22, 50 µM APV(42 µm), n = 21; 100 µM suramin (Srmn) (42 µm), n = 18; 4 µM alloxazine (Allx) (42 µm), n = 23; 1 µM TTX (42 µm), n = 20; 50 µM CBX (42 µm), n = 19; stimulated (126 µm), n = 24; 50 µM CBX(126 um), n = 27. Asterisk indicates p<0.05. Scale bars, 2 µm.

In experiments where stimulation was repeated every 5 minutes for 0.5 to 1 hour, the growth cones turned toward the region of activity (taking into consideration only those axons that extended more than 5 microns during the experiment) (Fig. 2a,b). Asymmetric filopodia extension preceded turning, with growth cones turning in the direction with more filopodia. We found that after high-frequency stimulation of the region to the right of a growth cone, the total perimeter of the growth cone membrane on the right side became greater than the left side, indicating preferential filopodia extension towards the source of activity (Fig. 2c–e). When a region of the culture was stimulated at a distance of 42 µm, 95% of growth cones (n = 20) had a greater surface perimeter on the right after the stimulation, compared to 47% of controls (n = 25), a statistically significant difference (p = 0.0022, Fisher's Exact Test). The attractive filopodia extension was blocked when cultures were pre-incubated in 1 µM TTX (n = 20, p>0.05) (Fig. 3c). It was also blocked by thapsigargin (1 µM) when the stimulation was performed at 42 µm (data not shown), indicating intracellular calcium stores are necessary mechanistically. We then increased the stimulation distance to 126 µm, a point from which multiple cell lengths would have to be traversed during guidance signalling. Under these conditions 83% of growth cones still showed directional extension (n = 24, p = 0.0322), an effect that persisted for up to 5 minutes.

### Biased filopodia extension is accompanied by VAMP-2 GFP redistribution

Using neurons transfected with GFP-tagged vesicle-associated membrane protein 2 (VAMP-2), we examined the cellular processes underlying the attractive behaviour. While VAMP-2 was homogeneously distributed throughout the growth cone prior to stimulation, it asymmetrically condensed to the membrane 1-2 minutes after the stimulation, with significantly more VAMP-2 on the side of the growth cone facing the stimulated region (p = 0.0344, student's unpaired t-test) (Fig. 3a,b and Video S2). This was not observed in the case of unstimulated controls(n = 21, p(L) = 0.4048, p(R) = 0.7723) (Fig. 3b).

### Mechanism of axonal attraction to activity involves calcium, glutamate and gap junctions, but not ATP or adenosine receptors

When the experiments were repeated in low (1 mM) extracellular Ca^2+^ from 42 µm, growth cones undergoing the stimulation protocol showed no directional bias, demonstrating the requirement of some optimal concentration of Ca^2+^ in the extracellular fluid (n = 20, p = 1.00, Fisher's Exact Test) (Fig. 3c).

To determine whether astrocytes were critical for guidance signal propagation, we tested whether blocking astrocyte-astrocyte communication would abolish the effect. Intercellular Ca^2+^ wave propagation is mediated largely by connexin-43-bridged gap junctions that form between astrocytes in confluent culture [28]. Carbenoxolone (CBX), a gap junction blocker, stops intercellular Ca^2+^ wave propagation without eliminating intracellular Ca^2+^ elevations in the astrocyte that is directly stimulated. We confirmed successful CBX (50 µM) inhibition of the intercellular waves using Fluo-4-AM imaging of spontaneous activity in our cultures (Video S3). Waves partially returned 10 minutes after drug washout (Video S4). In the presence of CBX (50 µM) the filopodia extension bias was observed at 42 µm (89%, n = 19, p = 0.0143) but not 126 µm (56%, n = 27, p = 1.00), suggesting Ca^2+^ wave transmission through astrocytes is necessary for the detection of activity over distances that span over multiple cell lengths.

Finally, we focused on the communication between astrocytes and growth cones by testing the involvement of the excitatory neurotransmitter glutamate and ATP, known to be released by astrocytes in response to depolarization [20]–[22], [28]–[30]. The competitive NMDA receptor antagonist D-2-amino-5-phosphonopentanoic acid (APV) or CNQX were bath applied during experiments performed at 42 µm. The biased extension was blocked with CNQX (10 µM) but not APV (50 µM). As shown in Fig. 3c, 86% of growth cones showed biased extension towards the right in the presence of APV (n = 21, p = 0.026), whereas CNQX treated growth cones were not statistically different from controls at 32% (n = 22, p = 0.2384). This shows the involvement of non-NMDA glutamate receptors. Fluo-4 imaging showed that glial waves could still be initiated in the presence of CNQX (data not shown). To test the involvement of ATP, experiments were also performed after bath application of suramin (100 µM), a broad spectrum antagonist at P2X and P2Y-purinergic receptors. Since ATP is metabolized by extracellular enzymes into adenosine [21], we also performed the assay in the presence of alloxazine (4 µM), an A2b receptor antagonist. The A2b receptor was selected for testing because previous work has shown it can regulate axonal growth cone behaviour [31]. Neither suramin nor alloxazine blocked the activity-dependent extension bias, with 83% (n = 18, p = 0.05) and 82% (n = 23; p = 0.02) biased towards the site of stimulation, respectively.

### Microglia do not play a role in the guidance mechanism

**Figure 4:**
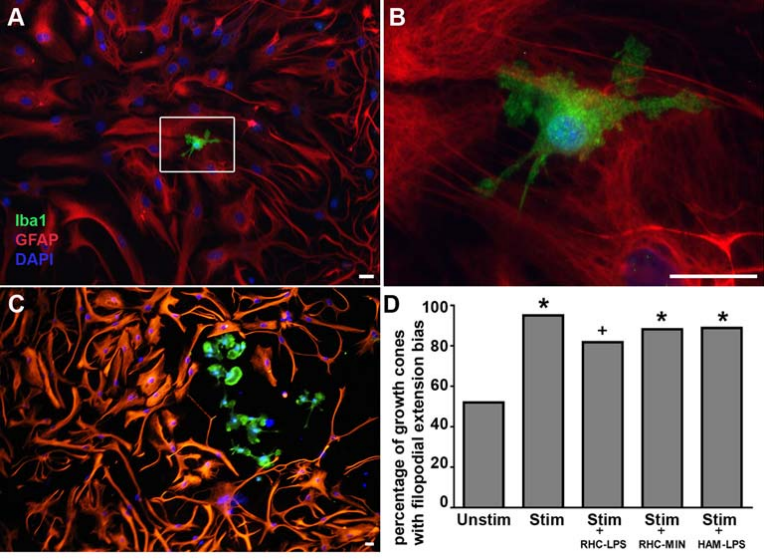
Activity-driven axon guidance in the presence of activated microglia and astrocytes. (a) Astrocytes and microglia in rat hippocampal cultures. Cultures were labeled using anti-Iba1 as a microglia marker (green), GFAP as an astrocyte marker (red), and were counterstained for nuclei with DAPI (blue). There were very few microglia in the cultures (∼1 in 300, see text). (b) shows an enlarged panel of (a). (c) The few microglia that were present in our cultures sometimes formed clusters. (d) The attractive filopodia extension bias occurs in the presence of conditioned media from minocycline treated rat hippocampal cultures (RHC-MIN) (n = 17) and LPS activated human adult microglia cultures (HAM-LPS) (n = 18). This was also the case for conditioned media from LPS (RHC-LPS) activated rat hippocampal microglia and astrocytes(n = 22). Asterisk indicates p<0.05, cross indicates p<0.06. Scale bars, 25 µm.

To test the involvement of microglia in this astrocyte-based guidance mechanism, we first characterized the presence of microglia in our dissociated hippocampal culture system. To quantify microglia in our cultures, Iba1 immunostaining was performed and DAPI (Sigma, Oakville, ON) was included during incubation with the secondary to determine the total number of cells in the culture. Iba1 expression is specific to microglia and macrophages, and does not cross react with neurons or astrocytes (Figure 4a,b). Images were captured from 20 different regions of each culture, for cultures from 6 different dissections. The approximate percentage of microglia was calculated to be 0.32%+/−0.093% (SEM) or about 1 for every 300 cells. Since the appearance of microglia in the cultures was rare it was therefore unlikely that microglia were consistently present in the vicinity of growth cones during the turning assays, making them an unlikely player in the mechanism. Also, the few microglia that were present tended to cluster in localized regions, possibly a remnant of the dissection process, or of regions where there were unhealthy cells (Figure 4c). This confined spatial distribution further decreased the probability of microglia involvement. Nonetheless, we also performed experiments in cultures that had been incubated in minocycline (10 µM) overnight and during the experiment. Minocycline is an antimicrobial tetracycline derivative that has been shown to inhibit the activity and expression of microglia-produced inflammatory mediators like nitric oxide (NO), interleukin (IL)-1ß, IL-6, tumour necrosis factor (TNF)-α, and nerve growth factor (NGF) [32]. Overall, 88% of growth cones showed the extension bias in the presence of minocycline (n = 17, p = 0.02) (Figure 4d). Together our experiments strongly suggest microglia activation is not involved in the glial wave guidance mechanism.

### Activity-driven axon guidance in the presence of pro-inflammatory factors

Glia are key modulators of axonal growth and regeneration following injury. Reactive microglia and astrocytes increase their production of a variety of cytokines that have been shown to inhibit axonal growth [6]–[8]. To address the question of whether these secreted factors would interfere with the glial wave guidance mechanism, we used two *in vitro* paradigms.

First, we activated astrocytes in the cultures by incubating them overnight in LPS (100 ng/mL) before performing turning experiments. This had no effect on the ability of the neurons to turn, as 82% of growth cones showed a bias toward the stimulus (n = 22; significant at p = 0.06) (Figure 4d).

Since the number of microglia in our cultures was relatively low, we wanted to more rigorously test this hypothesis. Microglia cells can be isolated from adult human brain tissue, and cultured for up to one week. When activated, these cultures are known to produce a number of cytokines including IL-1ß, IL-10 and TNF-α [33]. Conditioned media from LPS activated human adult microglia cultures (a kind gift from V. W. Yong, University of Calgary), was used in place of EBS during the growth cone asymmetry assay. Although some of the growth cones appeared less healthy (data not shown), the enriched activated microglia media did not diminish the turning (89%, a significant difference from unstimulated controls (n = 18; p = 0.05)) The results from both sets of experiments suggest the astrocyte wave guidance mechanism is still functional in the presence of reactive glia.

## Discussion

Taken together, our results suggest that growth cones turn in the direction of neuronal activity, and that this response is mediated by astrocytic Ca^2+^ waves. Additionally, this guidance signal involves the release of glutamate by astrocytes and activation of non-NMDA receptors on the growth cone. Since astrocytic Ca^2+^ wave transmission increases the distance from which neuronal activity can be detected, this mechanism of axon guidance could have a wide range of implications for the coordination of activity and neuronal connectivity in the nervous system. It may be important during development and for experience dependent or injury-related refinement of mature networks in certain areas of the brain.

### Attractive asymmetric filopodia extension precedes growth cone turning

Changes in the cytosolic calcium of filopodia initiate cascades of signalling events within the entire growth cone that can eventually result in attraction or repulsion [34], [35]. Although growth cone filopodia are very dynamic, our lab as well as others have shown that statistically distinguishable asymmetric distributions of growth cone filopodia are strongly correlated with a turning response in the direction with more filopodia [34]–[38]. Filopodia extension assays are particularly useful in situations where growth cones exist in complex environments that may not be permissive for extended axon outgrowth. For example, in our confluent DIV 14–24 cultures, neurite extension was limited simply by spatial constraints. Chemoattractive behaviour can thus be identified even when the growth cone is not undergoing active extension.

### Role of glutamate in inducing biased filopodia extension

Fluorometric assays show that astrocyte Ca^2+^ waves generate localized accumulations of glutamate up to concentrations of 100 µM. This glutamate release is regenerative, occurring in all subsequent cells that are involved in the Ca^2+^ wave. Waves of glutamate release travel at essentially the same speed as the Ca^2+^ wave itself [39]. Since biased growth cone filopodia extension was blocked by 10 µM CNQX in our experiments, it suggests glutamate mediates the attractive chemotaxic growth cone behaviour that is observed. It is important to note that the concentration of CNQX used here was too low to block the signalling event required for initiating Ca^2+^ elevation in the astrocyte. While glutamate does play a role in triggering Ca^2+^ influx in astrocytes, it achieves this result through metabotropic quisqualate receptors which are relatively CNQX insensitive and antagonized by 100 µM CNQX but not 10 µM CNQX in dissociated rat hippocampal cultures [19]. Consistent with the literature, we found that glial waves could still be evoked in the presence of 10 µM CNQX.

Since the AMPA receptor subunits GluR1 and GluR2 are expressed and organized into functional receptors in axonal growth cones of hippocampal neurons, glutamate probably acts directly on the growth cone at AMPA receptors [40]. Further studies are required to determine which specific non-NMDA receptor subtype is involved. Also, we cannot rule out the possibility that non-NMDA receptor activation on the axonal shaft located upstream of the growth cone triggers a signalling cascade that travels to the growth cone through the cytoplasm. It is interesting that non-NMDA receptors are uniquely involved, as it may provide functional separation from NMDA mediated signals, compartmentalizing the role of glutamate in neurotransmission, synaptic remodelling and axon guidance.

The inability of suramin or alloxazine to disrupt the turning effect given previous evidence of roles for ATP and adenosine in modulating axon guidance by regulating cAMP levels within growth cones [31], [41], suggests an interesting independence of this mechanism. However further investigation is necessary to fully determine if other adenosine receptor subtypes are involved in the signal transduction process.

Our observation that VAMP-2 redistributes asymmetrically to the membrane during the attractive filopodia extension towards activity further suggests a role for glutamate and non-NMDA receptors in the pathway. Compared to control neurons where VAMP-2 labeled synaptic vesicles are dispersed in the core of the growth cone, application of AMPA to cultured hippocampal neurons relocates VAMP-2 vesicles to the tips of growth cone filopodia [40], [42]–[43]. Synaptic vesicle exocytosis may provide the pathway necessary for activity-dependent delivery of AMPA receptor subunits to the surface [40]. An alternative role for VAMP-2 in this pathway is suggested by studies that have found SNARE proteins are involved in achieving asymmetric membrane expansion at the tip of growing neurites during attractive axon guidance [42], [43]. The asymmetric redistribution of VAMP-2 in this pathway corroborates evidence from our long-term turning studies that indicate asymmetric filopodia distribution is a mechanism for directional axon growth.

Previous studies have found that glutamate has either no effect or a repulsive chemotropic effect on isolated hippocampal axon growth cones depending on the concentration used [15]. These experiments were conducted by focally applying glutamate gradients on isolated neurons. To our knowledge, ours is the first study of the effect of glutamate on growth cone behaviour as it is released physiologically in response to remote activity. Our results do not contradict previous work because direct focal application of glutamate differs substantially from the release of glutamate by astrocytes. It is widely accepted that guidance cues have effects that are highly context-dependent [44]. Thus, in future studies it will be important to determine the spatial and temporal parameters which are critical for inducing the attractive effect seen here and to determine whether there are interactive effects of glutamate with other factors.

### Glial waves guide axons to activity

The present study suggests a role for glial waves in axon guidance. Astrocytes appear to amplify and translate signals about remote activity into local signals that can be used by cultured rat hippocampal neurons for axon pathfinding. It is because glia–to-glia Ca^2+^ wave transmission preserves neighbour relationships that directional growth towards activity is possible. Neuronal activity on its own would not be a robust directional cue because long and short range connections are involved and postsynaptic neurons are located in variety of spatial locations. Glutamate released by astrocytes is located more dispersely in the extracellular fluid, allowing it greater opportunity to interact with growth cones than synaptic glutamate which is tightly regulated. It is interesting to consider how astrocytes might balance calcium dependent glutamate release with their role in glutamate uptake, which is important for preventing neuronal excitotoxicity.

In these experiments, we were able to stop the propagation of intercellular glial Ca^2+^ waves by blocking gap junctions with carbenoxolone. While there is extensive evidence to support the role for gap junctions as a mechanism of glial wave propagation [45]–[47], other studies have found extracellular signalling is also involved in glial wave [48]. Evidence for a signalling role for ATP is particularly strong [29], particularly in light of evidence suggesting connexin blockers like carbenoxolone and heptanol may interfere with ATP dependent pathways [47].

Surprisingly, low Ca^2+^ extracellular bath solution was able to interfere with the mechanism even though a) astrocyte wave propagation relies on the release of intercellular Ca^2+^stores and, b) action potentials can still occur at 1 mM Ca^2+^ [47]. This suggests the requirement of some optimal extracellular calcium concentration for proper signal transduction within the growth cone. Previous studies have found that reduced extracellular Ca^2+^ concentrations suppress growth cone motility [49]. It is well-established that asymmetric Ca^2+^ transients are important in the regulation of growth cone filopodia motility and that the process is highly regulated [35].

Our results suggest the hypothesis that glial Ca^2+^ waves play a previously unrecognized role in intercellular signalling. It appears growth cones can detect the sequential passage of a glial Ca^2+^ wave from its activity-proximal side to its distal side, however the physical mechanism by which these small temporal and spatial gradients are detected by the growth cone is unknown. Unlike classical cues that are highly localized and regulated to ensure precision in spatial guidance, this activity-dependent, glial-based modulation is more dynamic. The transient nature of this cue is such that only repetitive activity could have a significant impact on axon guidance, which may allow growth cones to distinguish between signals and noise. Here we performed experiments with and without TTX (1 µM) to block sodium channels in neurons and found that neuronal action potential firing is required for initiation of astrocyte calcium waves in response to photoconductive stimulation. However, astrocytic Ca^2+^ waves also occur spontaneously *in vitro* and *in situ* [28], [50] so it is important to note that they may exert a guidance function in the absence of neuronal activity. Before we can speculate about how the attraction of growth cones towards activity may play a role in long term experience dependent axonal structural plasticity, further evidence from studies *in situ* and *in vivo* are required.

### Activated microglia do not inhibit activity driven axon guidance

After injury, axons must regenerate and navigate to their correct targets without the aid of cues and growth factors that were in place during development [8]. This difficult task is further complicated by the fact that trauma activates nearby microglia and astrocytes which release pro-inflammatory factors that impede axon outgrowth [8], [51]. Many approaches have been taken to aid the functional recovery of neuronal connections during axon regeneration in both the peripheral and central nervous system, ranging from the use of growth factors to electrical stimulation [52], [53].

Here we show that a glial wave based mechanism of axon guidance was able to provide directional instructions to growth cones in the presence of secreted factors from activated microglia and astrocytes. This presents the extremely exciting possibility that, in conjunction with other strategies, this methodology could be used to promote the regeneration of axons through similar environments following CNS injury *in vivo*. It also highlights the concern that the upregulation of glial waves could result in aberrant directional growth. This would also provide a potential therapeutic target for supporting axon regeneration.

## Materials and Methods

### Hippocampal Cell Culture

The hippocampi of newborn P0 Sprague-Dawley rats (Charles River, Wilmington, MA) were dissected, dissociated, and plated on p-type, 12 ohm/cm boron doped double side polished, 1 cm^2^ pre-cut silicon wafers, 1 mm in thickness (Silicon Quest International, Reno, NV) previously cleaned, oxidized, and coated with poly-D-lysine and laminin according to a published protocol[19]. All experimental protocols were approved by the University of Calgary Conjoint Faculties Research Ethics Board (Protocol #M05083). Dissociated primary rat hippocampal co-cultures were used at DIV 14–24, an age when astrocytes are grown to confluence and elaborate neuronal networks are established, but many growth cones persist.

### Human Adult Microglia Cell Culture and LPS activation

Microglia were obtained from human brain tissue with written consent from donors and approved by the University of Calgary Conjoint Faculties Research Ethics Board (Human Ethics Approval #17594), and were cultured according to a published protocol [33]. Microglia were plated at a density of 10 K per well in 96-well plates. Each well was filled with 10% fetal bovine serum containing medium. To activate microglia, lipopolysaccharide (LPS, 100 ng/mL) was added. The conditioned media from these wells was harvested 24 hours later, alongside control wells containing: media only, media+LPS, as well as microglia and media without LPS. Experiments were conducted directly in the media.

### Transfection

DIV 10–15 cultured rat hippocampal neurons were transfected with YFP-actin and GFP-VAMP-2 using Lipofectamine 2000 (Invitrogen, Burlington, ON) according to the manufacturer's instructions. Experiments were performed on these cultures during the week following transfection. YFPA was expressed throughout the lamellopodia and filopodia of growth cones, and in punctate form along neurites and the cell body. VAMP-2 was expressed throughout the growth cone and cell, particularly in association with the membrane. These expression patterns are consistent with those previously reported [24], [26], indicating over-expression probably did not interfere with the proteins' normal function.

### Photoconductive stimulation and electrophysiology

Photoconductive stimulation is a technique that stimulates action potentials in neurons that have been grown on silicon wafers [23], [24], [25]. Briefly, the silicon wafers are placed in a dish containing EBS and observed under an upright microscope. The dish contains two electrically isolated electrodes which apply current to the top and bottom of the silicon wafer. The region of neurons to be fired is selectively illuminated with the light from the microscope, and a two millisecond low-voltage current is applied across the wafer. This voltage step across the silicon-neuron junction induces action potentials by creating a capacitive transient in the neuron that activates voltage gated ion channels [54], [55]. To illustrate the efficacy of this technique, simultaneous electrophysiological recordings were made and can be seen in Supplementary Figure 1. Whole cell patch clamp recordings were performed in current clamp mode to record both spontaneous and induced action potentials, using an intracellular solution (pH 7.2; 300–310 mOsm), composed of 115 mM, 17.5 mM CsCl, 10 mM HEPES, 2 mM MgCl2, 10 mM EGTA, 4 mM ATP, 0.1 mM GTP.

### Live cell imaging of growth cone turning

Neuronal cultures were immersed in 24°C extracellular bath solution (EBS) during imaging [3.0 mM CaCl_2_, 2.0 mM MgCl_2_, 135 mM NaCl, 5.0 mM KCL_2_, 10 mM Glucose, 5.0 mM HEPES, ph 7.3, 315 mOsm]. Images were captured once every 5–20 seconds with a Watec 120N camera mounted on an Olympus BX61W1 microscope with an Umplanfi/IR 100× water immersion objective.

Axons of hippocampal cells were identified by their morphology. In our culture system, the axons are many times longer than dendrites, and are of comparably thin and constant diameter along their length with no spines and regular actin-rich variscosities along their length [1]. This method was determined to be ∼90% accurate as demonstrated by post-experiment immunostaining for the axonal marker tau-1 (Millipore, Billerica, MA). Growth cones were observed for 3–10 minutes prior to stimulation. During stimulation, 434 nm light was used and the aperture was partially closed to illuminate a 42 µm diameter region either 42 µm or 126 µm away from the right side of the growth cone. Under constant illumination, 30 2 ms pulses were delivered in 3 seconds, a protocol shown to be sufficient to induce action potentials in the neurons. Several frequencies of stimulation were tested in the 10–50 Hz range, and all were successful in producing the turning effect (data not shown). To limit photo-bleaching, the light was turned off between image captures. In the case of control experiments, the aperture was reduced, the stage moved, and the light shone to the right of the growth cone in identical fashion, but the electrical stimulus was not applied. Growth cones were monitored for 10 to 15 minutes following stimulation. Low calcium EBS, used to test calcium dependence, contained 1.0 mM CaCl_2_ and 4.0 mM MgCl_2_. All drugs were from Sigma-Aldrich (Oakville, ON). Drugs were dissolved in the recording EBS and cultures were pre-incubated in this solution for 10 minutes before experiments were performed.

### Calcium imaging

The Ca^2+^-sensitive fluorescent dye Fluo-4 AM (Invitrogen, Burlington, ON) was dissolved in DMSO to create a 3 mM stock solution. Cells were incubated in the dye, diluted to 5 µM with EBS, at 37°C for 30 minutes. After three washes in EBS the chips were used for experiments. Videos were captured with AMCap v9.11 (N.Danjou) at 7 frames per second.

### Immunocytochemistry

Cultures were fixed for 5 minutes in −20°C methanol (tau-1 staining) or for 15 minutes in −20°C acetone (Iba1 staining), and washed with PBS. This was followed by 1 hour of preblocking in 0.4% BSA, 0.1% Triton X-100, and 0.02% normal donkey serum in PBS (Sigma, Oakville, ON). Cultures were immunostained with the polyclonal antibody against Iba1 (Wako, Richmond, VA), monoclonal antibody against GFAP (Sigma, Oakville, ON), Tau-1 (Sigma, Oakville, ON) or NF160 (Millipore, Billerica, MA). These antibodies were detected by fluorescently conjugated secondaries raised in donkey (Chemicon, Temecula, CA).

### Data analysis and statistics

To identify asymmetric filopodia extension, the total perimeter of the left and right side of the growth cone was measured with ImageJ 1.34 s (W. Rasband, NIH) from each image captured during the course of the experiment. The central axis of the growth cone was defined by the angle of the trailing neurite. After normalizing the perimeters to the lowest and highest values for each side the data were divided into those taken ‘before stimulation’ and ‘after stimulation’. The designation of ‘right higher then left after stimulation’ was assigned to growth cones as indicated by the general statistics extracted from the distributions (mean, maximum, median, mode) (Graphpad Prism v4.0). The results are expressed as percentage of cases. A two-tailed Fisher's Exact Test was used. The standard alpha value of 0.05 was used to define statistical significance.

To obtain the trajectory plot for the long-term turning experiments, images were recorded every 1 minute for a ∼1 hr period. The center of the growth cone at the onset of the experiment was given a value of (0,0) where the original direction of growth was taken to be vertical. The position of the growth cone at subsequent time points was measured in ImageJ (W. Rasband), making sure the images were perfectly overlayed. The coordinates were then plotted on an X vs. Y plot of space.

To analyze intracellular VAMP-2 distribution, two frames from the experiment were selected for analysis: the first image from directly before the stimulation and the second image from a point around 1–3 minutes after the stimulation where protein condensation (appearing as bright spots) appeared the greatest. When no protein condensation was visible, the second image was selected at 3 minutes after stage translation. A region of interest (ROI) of consistent area was selected on the left membrane (L), right membrane (R), and cytoplasm (C) of each growth cone before and after stimulation. The mean grey value of the ROI was determined with ImageJ. The ratios L/C and R/C were taken to determine whether there was a difference in the cytoplasmic and membrane distribution of VAMP-2 and actin before and after stimulation. An unpaired two tailed Student's t-test was used to compare the means (alpha = 0.05).

## Supporting Information

Figure S1 Photoconductive stimulation induces action potentials in neurons. Shown is a current clamp recording of a DIV 14 hippocampal neuron in culture stimulated to fire action potentials at 5 Hz using photoconductive stimulation. Immediately following each elicited action potential, smaller EPSCs can be observed, a result of the initiation of local network activity. For comparison a final spontaneous action potential occurs in this recording at the end, as well as during the stimulation. Photoconductive stimulation very effective for inducing action potentials, a failure of the neurons to fire can be seen at the point marked by an asterisk. This allows visualization of the stimulation artifact, which is two milliseconds long and approximately 20 mV.
(7.05 MB TIF)

Video S1 This movie shows an astrocytic wave in a culture labeled with the calcium indicator fluo-4 AM after photoconductive stimulation at 10× (2 sec, 10 Hz). This video covers a period of ∼10 sec, presented here at 7 fps. The wave spreads out radially from an initial point of depolarization.
(1.51 MB MPG)

Video S2 This movie shows the redistribution of VAMP-2-GFP in an axonal growth cone after stimulation of a region to its right side. The video covers a total of ∼6 minutes (0.05 fps). VAMP-2 organizes near the membrane as filopodia extend in the direction of stimulation.
(7.57 MB MPG)

Video S3 This movie shows spontaneous activity in a culture labeled with the calcium indicator fluo-4 AM after treatment with the gap-junction blocker carbenoxolone (50 µM). This video covers a period of ∼2 min, presented here at 30 fps. There is very little spontaneous activity overall and no intercellular astrocyte calcium waves are observed.
(4.93 MB MPG)

Video S4 This movie shows the spontaneous activity of the same culture shown in Supplementary Video 3 about 10 minutes after the carbenoxolone (50 µM) has been washed out. This video covers a period of ∼3 min, presented here at 30 fps. Intercellular calcium waves are observed. The waves do not spread as far or as quickly as control cultures, suggesting residual gap junction blockage.
(7.37 MB MPG)
