# Supplementary figures and images for: Astrocytic Ca2+ Waves Guide CNS Growth Cones to Remote Regions of Neuronal Activity
Source: PLoS One. 2008 Nov 12;3(11):e3692. doi: 10.1371/journal.pone.0003692 (PMC2577300; doi:10.1371/journal.pone.0003692)

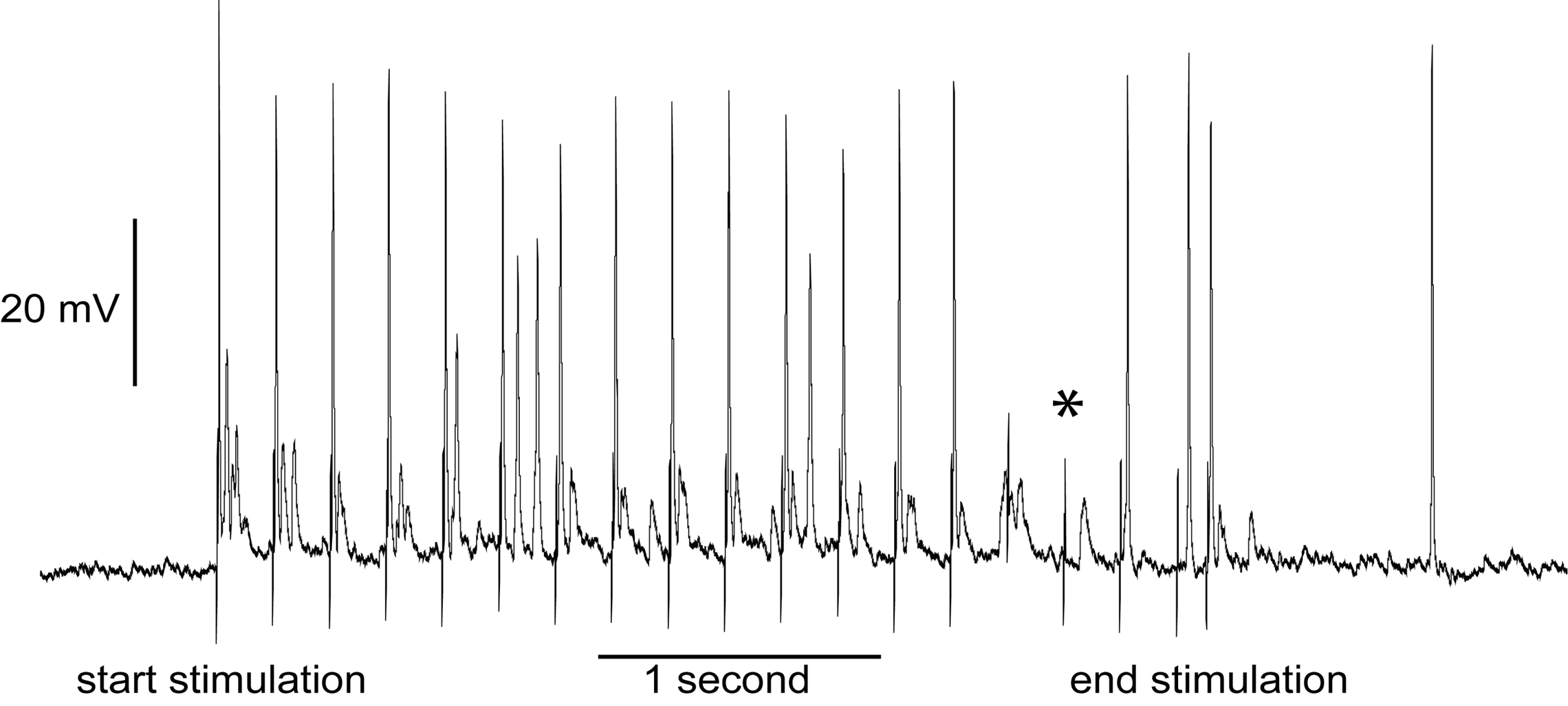

Supplement: Figure S1 — Photoconductive stimulation induces action potentials in neurons. Shown is a current clamp recording of a DIV 14 hippocampal neuron in culture stimulated to fire action potentials at 5 Hz using photoconductive stimulation. Immediately following each elicited action potential, smaller EPSCs can be observed, a result of the initiation of local network activity. For comparison a final spontaneous action potential occurs in this recording at the end, as well as during the stimulation. Photoconductive stimulation very effective for inducing action potentials, a failure of the neurons to fire can be seen at the point marked by an asterisk. This allows visualization of the stimulation artifact, which is two milliseconds long and approximately 20 mV. (7.05 MB TIF) [file pone.0003692.s001.tif]
